# Postmarketing safety surveillance data reveals protective effects of botulinum toxin injections against incident anxiety

**DOI:** 10.1038/s41598-021-03713-x

**Published:** 2021-12-21

**Authors:** M. Axel Wollmer, Tigran Makunts, Tillmann H. C. Krüger, Ruben Abagyan

**Affiliations:** 1 Asklepios Clinic North - Ochsenzoll, Asklepios Campus Hamburg, Medical Faculty, Semmelweis University, Hamburg, Germany; 2 Skaggs School of Pharmacy and Pharmaceutical Sciences, University of California San Diego, La Jolla, CA USA; 3 Oak Ridge Institute of Science and Education Fellowship at Office of Clinical Pharmacology, United States Food and Drug Administration, Oak Ridge, USA; 4 Hannover Medical School, Division of Clinical Psychology and Sexual Medicine, Department of Psychiatry, Social Psychiatry and Psychotherapy, Hannover, Germany

**Keywords:** Computational biology and bioinformatics, Medical research, Neurology

## Abstract

Randomized controlled trials (RCTs) have shown an antidepressant effect of glabellar botulinum toxin (BoNT) injections. In the FDA Adverse Event Reporting System (FAERS) database, BoNT injection is associated with reduced incidence rates of depression across various non-psychiatric indications, which confirms the previous findings independently of specific expectations to an antidepressant effect of BoNT. The rationale of using BoNT to treat depression is to interrupt proprioceptive body feedback that may reinforce negative emotions. Negative emotions also occur in other mental disorders, suggesting a transdiagnostic therapeutic potential of BoNT in psychiatry. Here we report an analysis of the FAERS database, in which we found that, compared to alternative treatments, BoNT injections were associated with lower incidence of anxiety symptoms and related disorders. Among seven indications/injection sites, we found this protective effect of BoNT in cosmetic use/facial muscles, migraine/facial and head muscles, spasms and spasticity/upper and lower limbs, torticollis and neck pain/neck muscles, and sialorrhea/parotid and submandibular glands (reporting odds ratios 0.79–0.27). These findings are encouraging for possible future RCTs on the use of BoNT as a treatment for anxiety and related disorders.

## Introduction

A series of randomized placebo-controlled trials (RCTs) and meta-analyses have shown that glabellar injections of botulinum toxin can reduce the symptoms of depression^1–6^. However, because the noticeable muscle relaxation induced by the toxin makes it impossible to truly blind the study participants for their group allocation, it is unclear to what extent a bias towards expectations/placebo effects in the treatment groups vs. disappointment/nocebo effects in the control groups may have inflated the large effect sizes observed in these trials.

To overcome this methodical limitation, we have reassessed the antidepressant action of botulinum toxin in the absence of specific expectations to that effect. For that purpose, we have gone into the FDA Adverse Event Reporting System (FAERS) and have compared the incidence rates of depression and related symptoms after treatment with botulinum toxin to a benchmark of alternative treatments for the same indications. Confirming and extending the results of the previous RCTs, we have found a significant preventive antidepressant effect of botulinum toxin across a broad spectrum of indications and injection sites^7,8^.

The rationale for the assessment of botulinum toxin as an antidepressant is the facial feedback hypothesis. The consequential idea that relaxing facial muscles expressing negative emotions would disrupt the proprioceptive afferences from these muscles and their maintaining and reinforcing effect on the expressed emotions^9^.

Since an excess of negative emotions is not specific for depression, but occurs in and determines the suffering associated with the majority of mental disorders, botulinum toxin therapy may not be specific for depression either, but may rather represent a transdiagnostic, emotion-focused treatment approach^10^.

Among the excessively experienced negative emotions, anxiety is one the most common.

Anxiety, panic and fear symptoms occur in many psychiatric conditions including depression, schizophrenia, borderline personality disorder, and anxiety disorders, in which panic, fear, or anxiety are the leading symptoms, are the most prevalent mental disorders of all^11^.

Proprioceptive and interoceptive signals are involved in the experience of panic, fear, and anxiety and in the pathophysiology of anxiety disorders. Conversely, relaxation and biofeedback techniques play a role in the treatment of these conditions^12–16^.

There is already one case series suggesting that glabellar injection of botulinum toxin may alleviate the symptoms of social anxiety disorder^17^. Accordingly, BoNT injections as a treatment of glabellar frown lines were associated with lower anxiety levels than other cosmetic treatments^18^. Moreover, in several studies on BoNT injections for indications like dystonia, facial spasms, chronic migraine, and hyperhidrosis, the treatment improved comorbid anxiety disorders or related symptoms, supporting the hypothesis that BoNT may have an anxiolytic effect^19–29^. Interestingly, anxiolytic effects of BoNT injections have also been observed in studies with mice and rats^30–32^. However, to date there are no RCTs investigating the effect of BoNT as a treatment for anxiety disorders. As in depression, expectations and blinding issues may affect the outcome of such trials in this indication^33,34^. Thus, before committing to any RCTs, we first analysed the FAERS database to investigate whether botulinum toxin injections may prevent incident anxiety symptomatology in patients who have *bona fide* no specific anticipation of such an effect.

## Methods

### FDA adverse event reporting system (FAERS)

FAERS database and its older version AERS store AE reports from healthcare professionals, patients, legal representativessubmitted through MedWatch^35^. If the reports are submitted to the manufacturer, the latter is mandated to evaluate and forward the reports to the FDA. This study used over fifteen million FAERS/AERS reports which, at the time of the analysis, included reports from January 2004 to March 2021. Reports were used to perform a retrospective inverse frequency analysis.

### Combining and normalizing FAERS/AERS data sets

Quarterly FAERS and AERS data sets, available as ASCII files online, were individually downloaded and separated in dollar-separated text (.txt) format. Since the data structure was not uniform in all quarterly files, it was necessary to modify the sets into a consistent table structure where missing fields were replaced with blank columns. Unix language/code was used for both data management restructuring, and analysis. Additionally, it was necessary to standardize all the drug names by generic terms due to the variability of brand names in the internationally submitted reports^8,36,37^.

### Cohort selection

**Figure 1:**
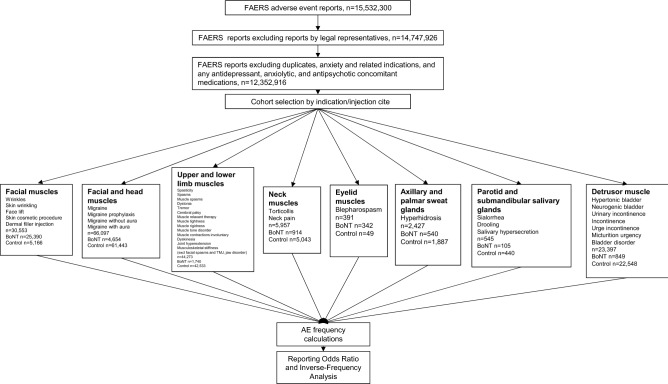
Analysis flow chart, and inclusion/exclusion terms for cohort selection, used in adverse event rate comparison between botulinum toxin and control cohorts.

**Figure 2:**
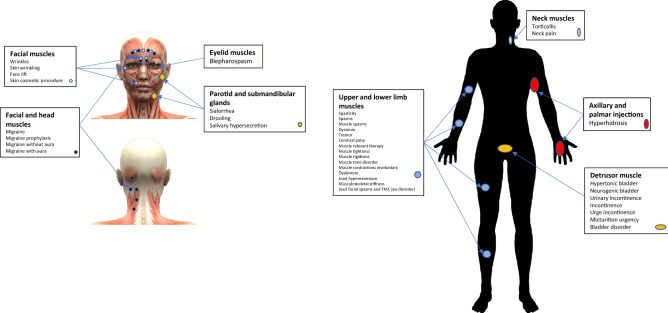
Study cohorts by indication and injection site. Christos Georghiou/shutterstock.com, decade3d—anatomy online/shutterstock.com.

A total of 15,532,300 unique reports until March 2021 were collected prior to the analysis. Reports submitted to the FDA by legal representatives were excluded to avoid potential bias. Additionally, reports related to patients taking both indicated and off-label antidepressants, anxiolytics, and antipsychotics along with reports where patients were comorbid with anxiety and related disorders (see details in S1-S3 Appendices) resulting in 12,352,916 reports. Cases with botulinum toxin (OnabotulinumtoxinA, AbobotulinumtoxinA, IncobotulinumtoxinA, and RimabotulinumtoxinB) were analysed to define eight indication and injection site cohorts (Figs. 1 and 2): (1) Cosmetic use—facial muscles (wrinkles, skin wrinkling, face lift, skin cosmetic procedure, dermal filler injection), n = 30,553; (2) Migraine—facial and head muscles (migraine, migraine prophylaxis, migraine without aura, migraine with aura), n = 66,097; (3) Spasms and Spasticity—upper and lower limbs (spasticity, muscle spasms, dystonia, tremor, cerebral palsy, muscle relaxant therapy, muscle tightness, muscle rigidness, muscle tone disorder, muscle contractions involuntary, dyskinesia, joint hyperextension, musculoskeletal stiffness), n = 44,273, disorders related to facial muscles such as facial spasms, temporomandibular joint disorder and jaw disorder were excluded; (4) Torticollis and neck pain—neck muscles, n = 5,957; (5) Blepharospasm—eyelid muscles, n = 391; (6) Hyperhidrosis—axilla and palm, n = 2427; (7) Sialorrhea—parotid and submandibular glands (drooling, salivary hypersecretion), n = 545; (8) Neurological and urinary bladder disorders—detrusor muscle (hypertonic bladder, neurogenic bladder, urinary incontinence, incontinence, urge incontinence, micturition urgency, bladder disorder), n = 23,397 (Figs. 1 and 2). The cohorts were separated into BoNT (exposed) and non-BoNT (control) sub-cohorts. Anxiety and related AE frequencies were calculated for patients in each sub-cohort and reporting odds ratios (RORs) were calculated to identify any protective effect through Inverse-Frequency Analysis.

### Statistical analysis

The statistical analysis of the FAERS and other safety surveillance data is well established, it includes frequencies, reporting odds ratios and 95% confidence intervals^38^. Below is the summary of the formulae.

**Figure 3:**
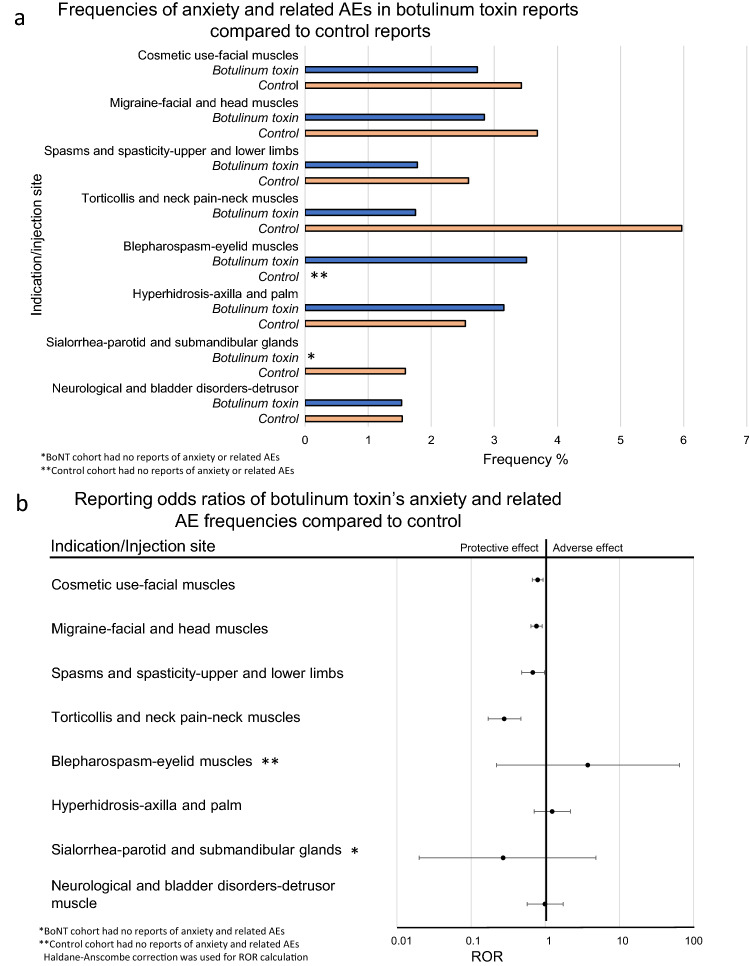
Frequencies and reporting odds ratios (ROR) of anxiety and related adverse events (AE). (**a**) Relative frequencies of anxiety events for patients administered botulinum toxin (BoNT) for various indications. (**b**) Reporting odds ratios with 95% confidence intervals (CI) as calculated by comparing frequencies of anxiety reports in patients administered botulinum toxin for each indication and respective control sub-cohorts.

1. Descriptive Statistics (Fig. 3a): Frequency for each side effect was calculated by the equation:
  $${\text{Reporting}}\;{\text{ frequency}} = {\text{No}}. \, \;{\text{of }}\;{\text{records }}\;{\text{with }}\;{\text{anxiety}}\;{\text{ and }}\;{\text{related }}\;{\text{AEs}}/{\text{No}}. \, \;{\text{of }}\;{\text{patient }}\;{\text{records}}$$
2. Comparative Statistics (Fig. 3b): Anxiety related report rates were compared via the Reporting Odds Ratio (ROR) using the following equations:
  $${\text{ROR }} = \, \left( {{\text{a }}/{\text{ b}}} \right) / \left( {{\text{c }}/{\text{ d}}} \right)$$

a = No. of anxiety and related AE reports in exposed group, b = No. in exposed group with no anxiety and related AE reports, c = No. anxiety and related AE reports in control group, d = No. in control group with no anxiety and related AE reports.

Standard Error (SE) of the LnROR value was calculated by the following equation:

$${\text{SELnROR }} = \, \surd \left( {{1}/{\text{a }} + { 1}/{\text{b }} + { 1}/{\text{c }} + { 1}/{\text{d}}} \right)$$

Error bars were computed using 95% confidence intervals.

$${95}\% {\text{ CI }} = {\text{ exp }}\left( {{\text{LnROR }} - { 1}.{\text{96}} \times {\text{SELnROR }}} \right){\text{ to exp }}\left( {{\text{LnROR }} + { 1}.{\text{96}} \times {\text{SELnROR }}} \right)$$

Haldane–Anscombe correction was used in small sample cohorts with zero reports of interest^39^.

## Results

### Botulinum toxin: anxiety and anxiety related adverse events

Patients who were administered BoNT had a significantly lower incidence of anxiety and anxiety-related AE reports, compared to the control groups. It was observed not only for cosmetic use in facial muscles (reporting odds ratios (ROR) 0.79, 95% confidence interval (CI) [0.67, 0.93]), but also for other indications and injection sites including:, migraine—facial and head muscles (0.76 [0.64, 0.91]), spasms and spasticity—upper and lower limbs, excluding facial muscles (0.68 [0.48, 0.98]), torticollis and neck pain—neck muscles (0.28 [0.17, 0.47]), There were no reports of anxiety or related AEs in the BoNT sialorrhea—parotid and submandibular glands sub-cohort. The reduced ROR value derived from 0/105 to 7/433 was evaluated as significant at 95% CI level, after the Haldane-Anscombe correction was applied (0.27 [0.020, 4.83]).

Almost no decrease in anxiety and related AE reports where BoNT was injected into the detrusor muscle in the neurological and urinary bladder disorders cohort was observed, but the reduced ROR value did not reach statistical significance (0.99 [0.57, 1.74]) and hyperhidrosis—axilla and palm cohort followed a similar trend (0.85 [0.51, 1.42]) (Fig. 3).

RORs for blepharospasm—eyelid muscles cohort exhibited increased potential risk, however not statistically significant (3.74 [0.22, 64.25]) (Fig. 3).

## Discussion

In this survey of the FAERS database we found that treatment with BoNT has a protective effect against incident anxiety disorders or symptoms. This effect was significant for the indications/injection sites cosmetic use/facial muscles, migraine/facial and head muscles, spasms and spasticity/upper and lower limbs, torticollis and neck pain/neck muscles, and sialorrhea/parotid and submandibular glands. There was no effect for hyperhidrosis/axilla and palm neurological and bladder disorders/detrusor muscle. With no reports in the control group, we found a numerically increased incidence of anxiety after BoNT injection in the blepharospasm/eyelid muscles indication. Although a bit less pronounced and consistent, these findings are largely in line with those from an analogous study on depression (ROR ranging from 0.13 to 0.60), supporting the potential of BoNT injections in the management of mental disorders^8^.

The evaluation of BoNT as a therapeutic for depression and other mental disorders associated with an excess of negative emotions was motivated by the facial feedback hypothesis^9^. However, the cumulating evidence of the efficacy of BoNT in such indications is not per se evidence of the accuracy of this rationale. Our previous study, which showed an antidepressant effect of BoNT across a broad range of indications and injection sites, opened up a broad spectrum of possible explanations for this effect^8,40^. Some of theses explanations are compatible with the facial feedback hypothesis while others challenge it. We have discussed these possibilities at length in the corresponding paper. In principle, they may also apply for our findings on anxiety. In the following, we will discuss them shortly in this regard.

As for modulation of facial feedback, as a mechanism of action, behind the observed effects on anxiety, it may explain the findings for cosmetic use and migraine. The corrugator muscles, which represent the key effectors in the facial expression of any emotions with negative valence, are the main site of BoNT injections in the cosmetic indication and are targeted in the migraine injection scheme, too. Raising the eyebrows belongs to the expression of anxiety and is accomplished by the frontalis muscle, which is also covered by the migraine scheme and is frequently injected for cosmetic reasons, too^41,42^. Hence, interruption of the corresponding proprioceptive feedback may explain the reduced incidence of anxiety. In blepharospasm, the numerically higher incidence of anxiety after BoNT treatment also fits into a similar concept. The main target in this indication is the orbicularis oculi muscle, which is involved in the expression of happiness (Duchenne’s smile) and narrows the palpebral fissure^43^. Its relaxation widens the eyes and may confer a negative shift in emotional expression and experience which, in turn, may promote anxiety. Of note, we observed a strong antidepressant effect of BoNT in the blepharospasm indication in our previous study with an overlapping population and an identical analytic approach^8^. Thus, BoNT injections around the eyes may have a differential effect on different psychiatric symptomatology. However, in the present study it is impossible to make a sharp distinction between the glabellar and orbital injections and their possibly opposite emotional effects, because the former is sometimes included in the treatment of blepharospasm and the latter may be injected in the cosmetic treatment of crow’s feet.

The reverberating interrelation between muscle activity and emotions is effective beyond the face^44^. Increased muscle tone in various body regions is a common phenomenon in anxiety disorders and may be both cause and effect of anxiety. In the treatment of anxiety disorders progressive muscle relaxation (PMR) is used to induce mental relaxation via tension and subsequent relaxation of skeletal muscles^12,15^. Proprioceptive afferences from the hypertonic musculature may account for the high prevalence of comorbid anxiety disorders or symptoms in patients suffering from dystonia or spasticity^14,19,23,25^. Accordingly, the anti-anxiety effect of BoNT injections in spasms and spasticity/upper and lower limbs as well as torticollis and neck pain/neck muscles may be explained by the interruption of these afferences^45^.

The body feedback concept may be extended to vegetative feedback mechanisms: hyperhidrosis is strongly associated with anxiety, and it is conceivable that increased sweating is not only a vegetative manifestation of anxiety but may also have an anxiety-enhancing feedback effect^46–48^. Botulinum toxin treatment has been successfully used as a treatment of anxiety disorders associated with hyperhidrosis^49^. However, we did not find a significant effect in our analyses. Bladder hyperactivity is also a vegetative correlate of anxiety, but we did not find association between BoNT treatment for this indication and decreased incidence of anxiety either^50^. As for saliva production, xerostomia is rather associated with anxiety than sialorrhea^51^. However, we found an association of BoNT treatment of sialorrhea with absence of anxiety. In summary, these findings do not support a role of interoceptive/vegetative feedback mechanisms in the observed anti-anxiety effect of BoNT.

It is possible, yet improbable that direct pharmacological BoNT effects within the CNS may explain its psychotropic action. BoNT may undergo targeted, transneuronal transport into the CNS where it may theoretically reach structures involved in the regulation of emotions^52,53^. In theory, BoNT may also reach the CNS and accomplish its anti-anxiety effect via systemic distribution. However, the amount of circulating BoNT may be very low, and the anti-anxiety effect shows no dose-dependence across the investigated indications with large vs. small muscles/muscle groups^54^. More likely, the peripheral action of BoNT may initiate a chain of neurochemical and neuroplastic changes that may be propagated to remote sites within the CNS^55^. Such neuronal reorganisation has been observed in patients treated for dystonia and spasticity^45,56–58^. It may also explain anxiolytic effects of BoNT applied at various injection sites in rats or mice^30–32^.

In the investigated indications, BoNT may have higher efficacy and better tolerability than the treatment options that were taken as comparators. Unfortunately, the FAERS database does not include efficacy data. As some of these conditions are chronic and burdensome, they may lead to secondary, reactive psychiatric comorbidities including anxiety disorders and related symptoms^59^. Hence, the more a treatment improves the primary condition for which it is given, the more it may also protect against the sequel of this condition. Thus, differential relief from the burden of disease between the BoNT and the control group may lead to overestimation of a possible specific anti-anxiety effect of BoNT. This may include relief from pain, which is a symptom of some of the investigated indications, especially migraine. However, superior efficacy and tolerability is not a unifying explanation of our findings either, because in the blepharospasm indication, in which it is the most effective treatment, there is no protective effect of BoNT against anxiety, but rather an anxiogenic tendency^60^. This also applies for the other indications in which BoNT did not show a protective effect against anxiety.

A neuronal structure that may mediate effects of BoNT on emotional experience and anxiety, is the amygdala^61–63^. Experimental studies have shown that facial injections of BoNT can modify its activity in response to emotional stimuli^64,65^.

There are some general limitations of this study. FAERS/AERS reporting is voluntary and often incomplete. Thus, the investigated data sets represent only a fraction of actual cases and the frequencies do not represent population incidences. Moreover, legal and scientific variables as well as newsworthiness may influence reporting to FAERS/AERS^66,67^. To address these limitations and to assess the significance of the difference between the sub-cohorts, we used disproportionality analysis with reporting odds ratios and 95% CI. Other limitations to consider include occasionally missing demographic variables, treatment doses and durations, and comprehensive medical records as well as bias associated with the comparator (differential efficacy, undetected differences between patients treated with the substance of interest and the comparator). Moreover, unreported life events and situations may have an imponderable impact on the incidence of anxiety. We excluded all the reports with comorbid anxiety disorders or anxiolytic medications (both labelled and off-label use); however, both may be underreported, which may affect the results. Exclusion of these reports may lead to underestimation of the efficacy of BoNT against anxiety, because we capture only preventive effects on incident anxiety. Therapeutic effects on prevalent anxiety may be more pronounced, but are not accessible to our analytic approach. Across all indications, there are differences in the concurrent medications between the BoNT and the reference groups, which may have confounding effects.

In conclusion, our findings show that BoNT administered for various indications and injection sites may have a protective effect against incident anxiety. The anti-anxiety effect represents an advantage over the alternative treatment options, because anxiety disorders and related symptoms are a frequent comorbidity in the respective indications. Even though afflicted with several limitations, our findings are encouraging to pursue the anxiolytic potential of BoNT in RCTs with patients suffering from anxiety disorders. Though there are effective pharmacological and psychotherapeutic treatments for these disorders, there is a need for further therapeutic options and BoNT may be one of them.

## Supplementary Information

Supplementary Information.

## Data Availability

The data sets are de-identified and made available to the public online by the United States Food and Drug Administration. Institutional Review Board requirements do not apply under 45 CFR 46.102. https://www.fda.gov/drugs/questions-and-answers-fdas-adverse-event-reporting-system-faers/fda-adverse-event-reporting-system-faers-latest-quarterly-data-files. Both FAERS and AERS datasets are de-identified and are made available online at: http://www.fda.gov/Drugs/GuidanceComplianceRegulatoryInformation/Surveillance/AdverseDrugEffects/ucm082193.htm. Institutional Review Board Requirements do not apply under 45 CFR 46.102. There was no direct human participation in the study. Thus, all experiments were performed in accordance with relevant guidelines and regulations.
