# Supplementary material for: Postmarketing safety surveillance data reveals protective effects of botulinum toxin injections against incident anxiety
Source: Sci Rep. 2021 Dec 21;11:24173. doi: 10.1038/s41598-021-03713-x (PMC8692576; doi:10.1038/s41598-021-03713-x)
Supplement: Supplementary file 1 — Supplementary Information. [file 41598_2021_3713_MOESM1_ESM.docx]

**Supplementary information**

**S1 Appendix. Antidepressant list (labeled and off-label-use)**

Sertraline, fluoxetine, citalopram, escitalopram, paroxetine, fluvoxamine, venlafaxine, desvenlafaxine, duloxetine, levomilnacipran , milnacipran, amitriptyline, amoxapine, clomipramine, desipramine, nortriptyline, doxepin, imipramine, protriptyline, trimipramine, maprotiline, bupropion, vilazodone, nefazodone,  trazodone, vortioxetine, mirtazapine, isocarboxazid, phenelzine, tranylcypromine, selegiline, milnacipran, tofenacin, etoperidone, amitriptylinoxide, dibenzepin, dimetacrine, dosulepin , adapin, lofepramine, melitracen, nitroxazepine, noxiptiline, pipofezine, butriptyline, demexiptiline, imipraminoxide, iprindole, metapramine, propizepine, quinupramine, mianserin, setiptiline, caroxazone, metralindole, moclobemide, pirindole, toloxatone, eprobemide, minaprine, ketamine.

**S2 Appendix. Anxiolytic and Antipsychotic (labeled and off-label-use)**

Estazolam, flurazepam, temazepam, triazolam, midazolam, alprazolam, chlordiazepoxide, clorazepate, diazepam, halazepam, lorazepam, oxazepam, prazepam, quazepam, clonazepam, pregabalin, gabapentin, buspirone, propranolol, hydroxyzine, trifluoperazine, olanzapine, quetiapine, aripiprazole, asenapine, cariprazine, clozapine, lurasidone, risperidone, ziprasidone, chlorpromazine, haloperidol, perphenazine, fluphenazine, brexpiprazole, iloperidone, loxapine, paliperidone, molindone.

**S3 Appendix Anxiety disorder and related indications/comorbidities**

MedDRA (24.0) applicable preferred terms based on “anxiety disorders and symptoms” Higher-Level Group Term (HLGT) and Higher-Level Term (HLT) classification.

HLT-Anxiety disorders NEC:

Anxiety disorder, anxiety disorder due to general medical condition, generalized anxiety disorder, neurosis, separation anxiety disorder.

HLT-anxiety symptoms:

Activation syndrome, agitation, akathisia, anticipatory anxiety, anxiety, nervousness, procedural anxiety, pseudoangina, stress, tension.

HLT-fear symptoms and phobic disorders:

Acrophobia, aerophobia, agoraphobia, aichmophobia, algophobia, animal phobia, astraphobia, autophobia, claustrophobia, cryophobia, dysmorphophobia, emetophobia, fear of (animals, closed spaces, death, disease, eating, falling, injection, open spaces, pregnancy, surgery, weight gain) fear related avoidance activities, glossophobia, haemophobia, haphephobia, hydrophobia, kinesiophobia, mysophobia, noctiphobia, nocturnal fear, nosocomophobia, nosophobia, ochlophobia, osmophobia, paruresis, performance fear, phagophobia, pharmacophobia, phobia, phobia of driving, phobia of exams, phobic avoidance, phonophobia, sitophobia, social anxiety disorder, social fear, thanatophobia, thermophobia.

HLT-obsessive-compulsive disorders and symptoms:

body dysmorphic disorders, compulsions, compulsive (check biting, hand washing, hoarding, lip biting, shopping), dermatillomania, dermatophagia, obsessive need of symmetry, obsessive rumination, obsessive-compulsive disorder, obsessive-compulsive symptom, olfactory reference syndrome, trichotemnomania, trichotillomania.

HLT-panic attacks and disorders:

Limited symptom panic attack, panic attack, panic disorder, panic reaction.

HLT-stress disorders

Acute stress disorder, anniversary reaction, burnout syndrome, catastrophic reaction, hyperarousal, post-traumatic stress disorder, Ulysses syndrome

**S4 Appendix. List of concurrent medications in BoNT and control groups**

| **Indication** | **BoNT cohort concurrent medications (Top 20 Reported)**  **% \| drug name** | **Control cohort medications**  **(Top 20 Reported)**  **% \| drug name** |
| --- | --- | --- |
| Cosmetic use | 0.22 \| Epinephrine,Lidocaine  0.24 \| Progesterone  0.24 \| Thyroid_porcine  0.24 \| radiesse (Calcium_hydroxyapetite)  0.25 \| Cetirizine  0.25 \| juvederm_ultra_xc (hyaluronic acid)  0.26 \| Bimatoprost  0.27 \| Estradiol  0.28 \| Atorvastatin  0.31 \| Lisinopril  0.39 \| Calcium  0.39 \| fish_oil  0.43 \| Ibuprofen  0.44 \| Aspirin  0.52 \| vitamins  0.55 \| Ergocalciferol  0.56 \| juvederm_voluma_xc (hyaluronic acid)  0.58 \| restylane (hyaluronic acid)  1.37 \| Levothyroxine  1.75 \| juvederm (hyaluronic_acid) | 2.28 \| Amlodipine  2.28 \| Metronidazole  2.28 \| Atorvastatin  2.38 \| Acetaminophen  2.50 \| Methotrexate  2.63 \| Butenafine  2.67 \| Ergocalciferol  2.69 \| Calcium  2.88 \| Omeprazole  2.98 \| Prednisone  3.02 \| Adalimumab  3.08 \| Avobenzone,Octocrylene,Triclosan  3.09 \| Adapalene,Benzoyl_peroxide  3.34 \| Clobetasol_propionate  3.46 \| Doxycycline  3.54 \| Brimonidine  4.13 \| Adapalene  4.40 \| Aspirin  5.20 \| Tretinoin  5.42 \| Levothyroxine |
| Migraine | 0.75 \| Cyclobenzaprine  0.80 \| Naproxen  0.89 \| Verapamil  0.90 \| Diclofenac  0.92 \| Ergocalciferol  0.92 \| Omeprazole  0.97 \| Fremanezumab  0.99 \| Aspirin  1.18 \| Acetaminophen,Butalbital,Caffeine  1.18 \| Magnesium  1.20 \| Eletriptan  1.29 \| Zolmitriptan  1.32 \| Acetaminophen  1.40 \| Ibuprofen  2.04 \| Levothyroxine  2.56 \| Rizatriptan  3.28 \| Galcanezumab  4.58 \| Topiramate  6.12 \| Sumatriptan  12.21 \| Erenumab | 1.29 \| Salbutamol  1.37 \| Acetaminophen,Butalbital,Caffeine  1.44 \| Omeprazole  1.68 \| Adalimumab  1.80 \| Aspirin  1.84 \| Diclofenac  1.85 \| Fremanezumab  2.35 \| Acetaminophen,Caffeine  2.65 \| Levothyroxine  2.70 \| Zolmitriptan  2.73 \| Naproxen  3.61 \| Acetaminophen  3.77 \| Acetaminop hen,Aspirin,Caffeine  3.73 \| Rizatriptan  4.17 \| Ibuprofen  4.30 \| Eletriptan  8.85 \| Galcanezumab  10.58 \| Topiramate  22.10 \| Sumatriptan  26.65 \| Erenumab |
| Spasms and spasticity | 1.15 \| Ibuprofen  1.15 \| Ketotifen  1.15 \| Lisinopril  1.21 \| Lansoprazole  1.21 \| Metoclopramide  1.26 \| Ipratropium_bromide  1.26 \| Methylprednisolone  1.38 \| Diphenhydramine  1.44 \| Dalfampridine  1.49 \| Ocrelizumab  1.61 \| Phenobarbital  1.72 \| Amlodipine  1.72 \| Ranitidine  1.78 \| Cetirizine  1.84 \| Levothyroxine  2.18 \| Valproic_acid  2.41 \| \| Aspirin  2.47 \| Acetaminophen  2.70 \| Tizanidine  9.77\| Baclofen | 2.22 \| Lisinopril  2.23 \| Pantoprazole  2.25 \| Metoprolol  2.27 \| Morphine  2.30 \| Furosemide  2.34 \| Topiramate  2.36 \| Amlodipine  2.40 \| Adalimumab  2.42 \| Quinine  2.52 \| Atorvastatin  2.92 \| evetiracetam  3.06 \| Ibuprofen  3.12 \| Levothyroxine  3.31 \| Omeprazole  3.75 \| Tizanidine  4.02 \| Acetaminophen  4.66 \| Aspirin  4.73 \| Cyclobenzaprine  11.11 \| Vigabatrin  46.32 \| Baclofen |
| Torticollis and neck pain | 0.77 \| Atenolol  0.77 \| Etizolam  0.77 \| Methocarbamol  0.77 \| Tizanidine  0.77 \| vitamins  0.77 \| VitD3  0.88 \| Baclofen  0.88 \| Epinephrine,Lidocaine  0.88 \| Lisinopril  0.88 \| Oxycodone  0.88 \| Simvastatin  0.88 \| Warfarin  0.99 \| Hydrochlorothiazide  0.99 \| Hydrocodone  0.99 \| Tramadol  1.20 \| Acetaminophen  1.20 \| Amlodipine  1.64 \| Metoprolol  1.86 \| Aspirin  2.52 \| Levothyroxine | 2.80 \| Atorvastatin  2.97 \| Amlodipine  3.47 \| Omeprazole  3.49 \| Epinephrine,Lidocaine  3.53 \| Acetaminophen,Oxycodone  3.77 \| Cyclobenzaprine  3.85 \| Hydrocodone  3.95 \| Morphine  4.32 \| Rofecoxib  4.48 \| Acetaminophen,Hydrocodone  4.68 \| Celecoxib  4.72 \| Levothyroxine  6.17 \| Tramadol  6.80 \| Aspirin  7.50 \| Naproxen  8.82 \| Oxycodone  8.88 \| Fentanyl  10.35 \| Acetaminophen  11.58 \| Ibuprofen  11.78 \| Diclofenac |
| Blepharospasm | 1.17 \| Calcium  1.17 \| Esomeprazole  1.17 \| Sodium hyaluronate  1.17 \| Hydrochlorothiazide,Triamterene  1.17 \| Omeprazole  1.17 \| Petrolatum  1.17 \| Ramipril  1.17 \| Salbutamol  1.17 \| VitC  1.17 \| VitE  1.46 \| Levothyroxine  1.75 \| Amlodipine  1.75 \| Lisinopril  2.05 \| Acetaminophen  2.05 \| Atenolol  2.05 \| Atorvastatin  2.05 \| Celecoxib  2.05 \| Ergocalciferol  2.05 \| Simvastatin  2.63 \| Cyclosporine  4.97 \| Aspirin | 2.04 \| Tafluprost  2.04 \| Timolol  2.04 \| Valproic_acid  2.04 \| Valsartan  2.04 \| Venetoclax  2.04 \| Multivitamins  2.04 \| Warfarin  2.04 \| Zolpidem  4.08 \| Acetaminophen  4.08 \| Azotesin drops  4.08 \| Cyclosporine  4.08 \| Etizolam  4.08 \| Ramipril  4.08 \| Glutathione drops  4.08 \| Teriflunomide  4.08 \| Trihexyphenidyl  6.12 \| Aspirin  6.12 \| Diphenhydramine  14.29 \| Prednisolone  16.33 \| Tetrabenazine  22.45 \| Carbamazepine |
| Hyperhidrosis | 0.56 \| Atorvastatin  0.56 \| Cefazolin  0.56 \| Cetirizine  0.56 \| Ergocalciferol  0.56 \| Spironolactone  0.74 \| Glycopyrronium  0.96 \| Antebate  0.96 \| Cefotiam  0.96 \| Chinese_medicine  0.96 \| Clobetasol_propionate  0.96 \| Mometasone  0.96 \| Heparinoid  0.96 \| Ibuprofen  0.96 \| Kindavate  0.96 \| Levocetirizine  0.96 \| Olopatadine  1.11 \| Valaciclovir  1.85 \| Amlodipine  1.85 \| Lisinopril  2.28 \| Levothyroxine | 1.38 \| Atorvastatin  1.38 \| Folic_acid  1.43 \| Metformin  1.48 \| Omeprazole  1.54 \| Ergocalciferol  1.54 \| Testosterone  1.59 \| Adalimumab  1.70 \| vitamins  1.75 \| Calcium  1.75 \| Ibuprofen  1.80 \| Oxybutynin  2.12 \| Aspirin  3.23 \| Acetaminophen  5.30 \| Conjugated_Equine_Estrogens, Medroxyprogesterone_acetate  5.72 \| Conjugated_Equine_Estrogens  5.99 \| Levothyroxine  7.58 \| Progesterone  14.47 \| Estradiol,Norethisterone  17.75 \| Glycopyrronium  30.79 \| Estradiol |
| Sialorrhea | 1.91 \| Levothyroxine  1.91 \| Lisinopril  1.91 \| Melatonin  1.91 \| Mosapride  1.91 \| Nitroglycerin  1.91 \| drysyrup  1.91 \| Pravastatin  1.91 \| Warfarin  2.86 \| Acetaminophen  2.86 \| Alendronic_acid  2.86 \| Carbamazepine  2.86 \| Droxidopa  2.86 \| Ergocalciferol  2.86 \| Finasteride  2.86 \| Midodrine  2.86 \| oyster  2.86 \| Multivitamins  3.81 \| Pimavanserin  3.81 \| Polyethylene_glycol  4.76 \| Metoprolol | 3.41 \| Metoprolol  3.64 \| Tiotropium  4.09 \| Omeprazole  4.32 \| hyoscine_hbr_hyt  4.32 \| Rasagiline  4.32 \| Salbutamol  4.55 \| Bendroflumethiazide  4.77 \| Amlodipine  5.00 \| Carbidopa,Levodopa  5.23 \| Fluticasone  5.23 \| Pramipexole  5.46 \| Docusate  5.46 \| Valproic_acid  6.36 \| Acetaminophen  7.05 \| Levothyroxine  7.95 \| Atropine  8.18 \| Aspirin  8.18 \| Lansoprazole  14.55 \| Glycopyrronium  45.00 \| Scopolamine |
| Neurological and urinary bladder disorders | 1.30 \| Apixaban  1.30 \| Fesoterodine  1.30 \| Prednisone  1.30 \| Promethazine  1.30 \| Trospium  1.41 \| Dalfampridine  1.41 \| Ocrelizumab  1.65 \| Cyanocobalamin  1.65 \| Epinephrine,Lidocaine  1.77 \| Omeprazole  1.77 \| Valsartan  1.89 \| Metoprolol  1.89 \| Natalizumab  2.00 \| Amlodipine  2.36 \| VitD3  2.59 \| Levothyroxine  2.83 \| Oxybutynin  3.18 \| Solifenacin  3.30 \| Aspirin  4.01 \| Mirabegron | 2.49 \| Pantoprazole  2.50 \| Losartan  3.44 \| Furosemide  3.46 \| Acetaminophen  3.57 \| Lisinopril  3.65 \| Simvastatin  3.76 \| Metformin  4.14 \| Omeprazole  4.14 \| Metoprolol  4.98 \| Atorvastatin  5.48 \| Darifenacin  5.56 \| Amlodipine  6.41 \| Levothyroxine  7.82 \| Aspirin  7.85 \| Tamsulosin  10.33 \| Fesoterodine  11.20 \| Tolterodine  15.62 \| Oxybutynin  21.79 \| Mirabegron  22.46 \| Solifenacin |
